# Sporopollenin Microcapsule: Sunscreen Delivery System with Photoprotective Properties

**DOI:** 10.3390/pharmaceutics14102041

**Published:** 2022-09-24

**Authors:** Silvia Tampucci, Giorgio Tofani, Patrizia Chetoni, Mariacristina Di Gangi, Andrea Mezzetta, Valentina Paganini, Susi Burgalassi, Christian Silvio Pomelli, Daniela Monti

**Affiliations:** 1 Department of Pharmacy, University of Pisa, Via Bonanno 6, 56126 Pisa, Italy; 2 Department of Physics, University of Pisa, Largo B. Pontecorvo 3, 56127 Pisa, Italy

**Keywords:** photoprotection, sporopollenin, sunscreen, ethylhexyl triazone, skin delivery

## Abstract

In recent years, the demand for high-quality solar products that combine high efficacy with environmentally friendly characteristics has increased. Among the coral-safe sunscreens, ethylhexyl triazone (Uvinul^®^ T150) is an effective organic UVB filter, photostable and practically insoluble in water, therefore difficult to be formulated in water-based products. Oil-free sunscreens are considered ideal for most skin types, as they are not comedogenic and do not leave the skin feeling greasy. Recent studies reported that pollen grains might represent innovative drug delivery systems for their ability to encapsulate and release active ingredients in a controlled manner. Before being used, the pollen grains must be treated to remove cellular material and biomolecules, which could cause allergic reactions in predisposed subjects; the obtained hollow structures possess uniform diameter and a rigid wall with openings that allow them to be filled with bioactive substances. In the present work, pollen from *Lycopodium clavatum* has been investigated both as a delivery system for ethylhexyl triazone and as an active ingredient by evaluating its photoprotective capacity. The goal is to obtain environmentally friendly solar aqueous formulations that take advantage of both sunscreen and sporopollenin microcapsules’ UV protection with a relatively low cost, as these pollen grains are widely available.

## 1. Introduction

Sunscreens play a strategic role in the prevention of photo-induced damage. In recent years, the demand for high-quality solar products that combine high efficacy with excellent sensory properties, but are also environmentally friendly, has increased [1]. In particular, the impact of solar products on marine ecosystems with the possibility of bioaccumulation of organic filters and consequent toxicity towards the coral reef has received attention [2,3,4].

Several strategies can be adopted to limit the environmental damage caused by UV filters. Among these, the use of natural photoprotectors in association with UV filters in solar products has been proposed for an increase in solar protection factor (SPF) combined with an antioxidant activity, which therefore allows reduction in the amount of chemical filters in solar formulations [5]. Moreover, the formulation also has a role, as the different excipients employed contribute to some ecotoxicological hazards [1].

In the last decade, the research in drug delivery systems deriving from substances of natural origin has been intensified and pollen grains have attracted a lot of interest as microencapsulation materials due to their ecological nature, uniform size distribution in the micrometric scale and chemical–physical stability [6,7,8,9,10,11,12,13]. Indeed, the outermost layer (exine) of the pollen grain wall is mainly composed of sporopollenin, an extremely robust mixture of biopolymers resistant to physical, biological and chemical non-oxidative degradation. Conversely, the innermost layer (intina) of the pollen grain walls consists of cellulose, pectin, proteins and polysaccharides susceptible to acidic hydrolysis and alkaline treatments [7]. Due to their robustness, sporopollenin microcapsules (SPMs) have been proposed for various applications, including drug delivery [8,14,15]. SPMs with different sizes and morphology can be obtained from a variety of plants, but even though many recent studies have demonstrated the capability of SPMs to control and enhance the delivery of drugs [16,17,18], only a few of them concern cutaneous and cosmetical applications [19,20].

Among the different plant sources that have been investigated throughout the years, SPMs from *Lycopodium clavatum* have been shown to possess a uniform diameter of about 25 μm [21] and to be endowed with antioxidant properties [22]. Furthermore, *L. clavatum* pollen grains are easily available and not expensive, therefore representing an ideal material for new excipient research. 

In the present work, pollen from *L. clavatum* has been investigated as a delivery system for the organic solar filter ethylhexyl triazone (ETZ, Uvinul^®^ T150), which is authorized for use in Europe up to a 5% concentration (Annex VI, Regulation (EC) No. 1223/2009) and is considered environmentally friendly [1] and reef-safe [23]. A particularly interesting property is its high molecular weight (823.07 g mol^−1^), which impairs skin penetration. It shows a broad absorption profile in the UVB region and it is considered not only highly photostable, but it acts as a photostabilizer for other organic filters such as avobenzone [24,25]. The water insolubility makes this filter easily embeddable in water-resistant formulations, while the formulation in aqueous media presents some difficulties. On the other hand, oil-free sunscreens are considered ideal for most skin types, as they are not comedogenic and do not leave the skin feeling greasy; in particular, for people prone to acne it is recommended to avoid oil-based formulations [26]. 

The aim of the present work is to obtain an aqueous formulation containing ETZ encapsulated in the hydrophobic cavity of *L. clavatum*, which should be able to deliver the solar filter to the first layers of the skin, in order to exploit the solar filter substantivity (i.e., the capability to adhere well to the skin, without being easily removed by simple washing; [27]). The SPMs were extracted from *L. clavatum* pollen grains through a standardized protocol that includes alkaline and acidolysis steps and the obtained microcapsules have been characterized through different methods. Moreover, the capability of SPMs to act as a photoprotective agent has been investigated, to take advantage both of the sunscreen and sporopollenin microcapsules’ UV protection. Finally, the in vitro permeation and penetration through and into the skin layers after cutaneous application of an aqueous dispersion of ETZ-SPMs were studied using porcine ear skin as a model.

## 2. Materials and Methods

### 2.1. Materials 

The following materials were used: pollen from *Lycopodium clavatum* L. (Merk Life Science S.r.l., Milano, Italy); ethylhexyl triazone (ETZ, Uvinul^®^ T150; BASF, Ludwigshafen, Germany); polyoxyethylene-20-oleylether (Brij98, Sigma Aldrich (Darmstadt, Germany); xanthan gum (Xantural 75; CP Kelko, Atlanta, GA, USA).

All other chemicals and solvents were of analytical grade. Ultrapure water was obtained using MilliQ^®^ plus apparatus (Millipore, Milan, Italy).

### 2.2. Extraction of Sporopollenin of Lycopodium Pollen

*Lycopodium clavatum* pollen grains (100 g) were refluxed in 500 mL of acetone for 6 h, and then the solution was filtered under vacuum with glass fiber filters and air-dried overnight. Pollen grains were refluxed in 500 mL of KOH (6% *w/v*) for 6 h, filtered under vacuum, rinsed with distilled water (5 × 500 mL) and with hot ethanol (500 mL) and air-dried overnight.

Subsequently, the pollen grains were treated with 500 mL phosphoric acid (85% *v/v*) at 70 °C for 6 h, filtered under vacuum, rinsed with hot water (5 × 800 mL), acetone (600 mL), hydrochloric acid 2 M (600 mL), sodium hydroxide 2 M (600 mL) and washed again with hot water (5 × 500 mL), acetone (600 mL) and ethanol (600 mL).

Finally, the SPMs obtained were air-dried until reaching 30.0 g weight. 

### 2.3. Preparation of Encapsulated ETZ-Loaded Sporopollenin Microcapsules (ETZ-SPMs)

Preliminary studies for the selection of the best method for encapsulation of ETZ inside sporopollenin microcapsules have been performed, varying both the solvent in which to solubilize the sunscreen and the treatment to eliminate the non-incorporated ETZ fraction. In all cases, the passive loading technique was employed and finally acetone was selected as the best solvent.

Briefly, 150 mg of ETZ was solubilized in 4 mL of acetone and subsequently an exactly weighed amount (300 mg) of sporopollenin microcapsules was added, vortexed for 7 min and stirred at 4 °C for 2 h. To remove the non-incorporated ETZ fraction, the suspension was subjected to centrifugation at 4000 rpm for 7 min (an additional 2 mL was used to wash the vial to recover all the material).

For further removal of the non-encapsulated ETZ from the recovered microcapsules, the samples were subjected to 3 more consecutive cycles of solvent addition and centrifugation (6 mL acetone and 4000 rpm for 7 min). For each cycle, the supernatant was collected and analyzed by spectrophotometry.

Finally, the residue, representing the ETZ-loaded sporopollenin microcapsules (ETZ-SPMs), was collected and dried at 37 °C until a constant weight was reached. The amount of sunscreen encapsulated was spectrophotometrically determined. In order to control the reproducibility of the production method, six different batches were prepared.

### 2.4. Encapsulation Efficiency

The quantitative determination of ETZ encapsulated into the sporopollenin microcapsules was performed with a UV–Vis spectrophotometer (UV-2600 Shimadzu Europe GmbH, Milan, Italy) for comparison with an external calibration curve (R^2^ = 0.998) at the maximum absorption wavelength of 315 nm.

Basically, to ensure complete release of the incorporated solar filter, 5 mg ETZ-SPMs were suspended in 10 mL ethanol. The suspension was stirred for 30 min at room temperature and then centrifuged at 4000 rpm for 7 min. The supernatant was collected and analyzed. 

The entrapment efficiency of ETZ was calculated using the following equation:

$$Entrapment\;efficiency,EE\%=\frac{mass\;of\;solar\;filter\;in\;the\;microcapsule}{mass\;of\;solar\;filter\;added\;to\;the\;microcapsule}\times 100.$$

### 2.5. FTIR Spectral Analysis and Imaging Techniques

ATR-FTIR spectra of *Lycopodium clavatum *L. untreated pollen grains, SPMs, ETZ and ETZ-SPMs were recorded with an IR Cary 660 FTIR spectrometer (Agilent Technologies Santa Clara, CA, USA) using a macro-ATR accessory with a Zn/Se crystal. The spectra were measured as previously described by [9] in a range from 4000 to 500 cm^−1^, with 32 scans both for background and samples. Chemical imaging data were collected by Agilent’s ATR-imaging technique using an FTIR Cary 620 imaging system equipped with a 64 × 64 focal plane array (FPA) detector cooled by liquid nitrogen and a Ge crystal. Spectra were measured, from 3300 to 900 cm^−1^, with 256 scans for both background and pollen samples. For all the FTIR analyses, a few milligrams of sample were used.

The same samples were also observed with the MicroStar120 optical microscope, (Reichert-Jung, Buffalo, NY, USA) at 40× magnification.

### 2.6. Differential Scanning Calorimetry

The thermal behavior of ETZ, SPMs, ETZ-SPMs was analyzed by a differential scanning calorimeter (DSC 4000, Perkin Elmer, Milan, Italy) following the procedure reported in [28]. The method involves two steps: the sample was maintained at 10 °C for 1 min and then exposed to heating from 10 °C to 300 °C with a constant heating rate of 10 °C/min under nitrogen atmosphere (20 mL/min).

### 2.7. Thermogravimetric Analysis

The thermal stability was investigated by thermal gravimetric analysis (TG) (TA Instruments Q500 TGA, New Castle, USA, Delaware; weighing precision ± 0.01%, sensitivity 0.1 µg, baseline dynamic drift < 50 µg). The temperature calibration was performed using the Curie point of nickel and Alumel standards and for mass calibration weight standards of 1 g, 500 mg and 100 mg were used. All the standards were supplied by TA Instruments Inc. In a platinum crucible as a sample holder, 12–15 mg of each sample was heated. First, the heating mode was set to isothermal at 60 °C in N_2_ (80 cm^3^/min) for 30 min. Then, the sample was heated from 40 °C to 500 °C at 10 °C/min under nitrogen (80 cm^3^/min) and maintained at 800 °C for 3 min. Mass change was recorded as a function of temperature and time. TGA experiments were carried out in duplicate.

### 2.8. In Vitro Release Study

In vitro release studies through a dialysis membrane (regenerated cellulose, Spectra/Por molecular porous membrane tubing, 12–14 KDa; Spectrum Laboratories, Inc., Piscataway, NJ, USA) were performed with Gummer-type diffusion cells.

Briefly, 200 mg of ETZ-SPMs was added in the donor compartment in direct contact with the membrane. The receptor compartment consisted of 5.0 mL of pH 7.4 (phosphate buffered saline, PBS) added to 0.01% Brij 98 maintained at 37 °C and stirred at 600 rpm. Brij 98 was added to improve the ETZ solubility in the receiving fluid [29].

At predetermined time intervals, 5 mL of the samples was withdrawn and replaced with the same amount of fresh receiving fluid. All the experiments lasted 24 h and were performed in triplicate. The amount of ETZ in the samples was determined by HPLC after filtration through cellulose acetate filters (0.22 um pore size, Minisart^®^ NML Syringe filters, Sartorius, Florence, Italy).

### 2.9. Sun Protection Factor (SPF) Determination

The SPF quantifies the filtering capacity of a sun product, providing a correct indication of the level of protection and therefore of the duration of exposure to UV rays without having erythematogenic effects.

The sun protection factor was determined using the Labsphere 2000S spectrophotometer (Labsphere UV-2000S UV Transmittance Analyzer, Labsphere, Inc., North Sutton, NH 03260, USA) and the substrate used for the determination of the SPF was surgical tape (3M™ Health Care, St, Paul, MN, USA).

Briefly, an exactly weighed amount of the sample was uniformly applied with a latex glove-coated finger on the Transpore™ membrane to obtain a 2 mg/cm^2^ product/surface ratio. Then, it was allowed to rest for 15 min at room temperature, protected from light. 

For the SPF determination, an aqueous dispersion of xanthan gum 0.8% *w/w* was prepared and added to an appropriate amount of ETZ-SPMs corresponding to ETZ 1.0% *w/w*. As a reference, both SPMs and natural pollen grains in the same semisolid formulation were analyzed.

Before reading the substrate on which the solar product was spread, the substrate was read as it was (blank scan), 12 scans were carried out.

The estimated value of the SPF is:

**Rated SPF = SPF − E**,

where E is the error that depends on the number of measurements (scans) collected.

The absorbance for the two radiations was calculated as follows:

$$a\;\mathrm{UVA}=\frac{1\mathrm{nm}}{\left(400\;\mathrm{nm}-320\;\mathrm{nm}\right)}\;(\frac{A_{320}}{2}+A_{321}+A_{322}+\ldots +A_{399}+\frac{A_{400}}{2}),$$

$$a\;\mathrm{UVB}=\frac{1\;\mathrm{nm}}{\left(320\;\mathrm{nm}-290\;\mathrm{nm}\right)}(\frac{A_{290}}{2}+A_{291}+A_{292}+\ldots +A_{319}+\frac{A_{320}}{2}).$$

The *Boots Star Rating* and the *Star Category* are based on the *UVA ratio* parameter and classified as reported in Table 1:

$$\mathrm{Ratio}\;\mathrm{UVA}=\frac{a\;UVA}{a\;UVB}$$

### 2.10. In Vitro Cutaneous Permeation and Distribution Studies

Porcine ear skin, used as a model, was obtained from freshly sacrificed animals in a local slaughterhouse and treated as described in [30]. The pig hairs were abscised and the skin (thickness: 1.46 ± 0.06 mm and area available for permeation: 1.23 ± 0.99 cm^2^) was placed in the Gummer-type diffusion cells.

The donor phase consisted of 50 mg (or 200 mg) of ETZ-SPMs added to 100 μL (or 400 μL) of PBS + 0.01% Brij 98 in order to improve the contact of the formulation with the skin. 

The receiving phase consisted of pH 7.4 (phosphate buffered saline, PBS) added to 0.01% Brij 98 maintained at 37 °C and stirred at 600 rpm.

At predetermined time intervals, the receiving phase (5 mL) was withdrawn for HPLC analysis and replaced with the same volume of fresh fluid, and sink conditions were maintained throughout the entire study. All experiments lasted 24 h and were replicated four times.

At the end of the permeation experiments, the skin was collected and treated following the procedure reported and validated in [31]. 

Briefly, at the end of the in vitro permeation experiments the skin was removed from the diffusion cells, rinsed with distilled water to eliminate excess formulation from the skin surface and gently wiped with cotton-wool wipes.

Afterwards, the stratum corneum was removed using the tape-stripping method as described in [31]. The skin was stripped using an adhesive tape (Tesa film N. 5529; Kristall-Klar, Beiersdorf, Hamburg, Germany) and the tape strips were pressed on the skin by applying uniform pressure in order to obtain intimate contact between the film and the skin. The first tape strip was discarded, as this represents unabsorbed material only. Then, the subsequent strips were carefully removed and the entire procedure was repeated 25 times (tape strips no. 1–25). The strips were collected following a predetermined scheme in seven glass vials, each containing 5 mL of ethanol, sonicated for 10 min and subjected to centrifugation (15 min, 4000 rpm). The supernatant was collected for HPLC analysis to determine the amount of ETZ in the SC. To evaluate the presence of ETZ in the viable epidermis, the samples were treated with 2 mL of 2% sodium dodecyl sulfate (SDS) for 23 h under magnetic stirring and then 4 mL of methanol was added and stirred for 1 h. 

The mixture was centrifuged at 4000 rpm for 15 min and then supernatant was collected for HPLC analysis.

### 2.11. HPLC Analysis

The quantitative determination of ETZ in the receiving fluid and in skin samples was determined by a reverse-phase HPLC method with apparatus consisting of an LC-10AD pump and 20 μL Rheodyne injector, SPD-10AV detector and C-R4A computer integrating system (Shimadzu Corp., Kyoto, Japan). A reverse-phase C18 column (Synergi 4u Fusion-RP 80A, 150 × 4.6 mm, Phenomenex, Torrance, CA, USA) was used. Isocratic elution was performed using a mobile phase consisting of a mixture of CH_3_OH:H_2_O acidified with 1% glacial acetic acid (98:2 *v/v*), filtered through a 0.45 μm pore size membrane filter. The detection wavelength was 310 nm, the flux was 1.0 mL/min and the retention time was 11.3 min.

The amount of ETZ in the samples was determined by comparison with appropriate external standard curves obtained applying the least square linear regression analysis. For in vitro studies, the calibration curves were obtained by dissolving the ETZ in acetonitrile and then diluting with PBS pH 7.4 added to 0.01% Brij 98. In the case of biological materials, a standard curve was obtained by adding increasing amounts of ETZ to a blank biological matrix.

### 2.12. Statistical Analysis

Statistical differences in the exploited SPF between empty and ETZ-loaded SPMs were evaluated applying Student’s two-tailed unpaired *t*-test (GraphPad Prism Software, version 8.4.3, San Diego, CA, USA).

In the case of cutaneous permeation and distribution studies, statistical differences between the different doses of product applied on the skin have been determined by Student’s two-tailed unpaired *t*-test using GraphPad Prism software, version 8.0. 

The evaluation included calculation of the mean and standard error (S.E). In all cases, differences were considered statistically significant at *p* < 0.05.

## 3. Results and Discussion

### 3.1. Preparation of Encapsulated ETZ-Loaded Sporopollenin Microcapsules (ETZ-SPMs)

To verify that the sporopollenin microcapsules obtained after the extraction procedure can be used as a vehicle for the skin application of a sunscreen, the passive loading method was employed to encapsulate the solar filter ETZ, after its solubilization in acetone.

The results obtained are reported in Table 2 in terms of the amount of ETZ encapsulated by grams of SPMs (ETZ, mg/g_SPMs_) and EE%, for the different batches prepared. The prepared ETZ-SPMs exhibited an inter-batch variability with a CV% < 20, that can be considered acceptable for an attempt on a laboratory scale, which will in any case be subjected to further refinements. UV–Vis analysis of ETZ-SPMs showed ETZ loading content in the range 36.99–62.12 mg for grams of sporopollenin microcapsules and encapsulation efficiency in the range 6.49–11.91, suggesting that *L. clavatum* sporopollenin microcapsules may represent a potential system for the encapsulation of active substances. 

### 3.2. FTIR Spectra Analysis and Optical Microscopy

*Lycopodium clavatum *L. untreated pollen grains, SPMs, ETZ and ETZ-SPMs were analyzed by infrared spectroscopy and FTIR infrared microscopy.

**Figure 1:**
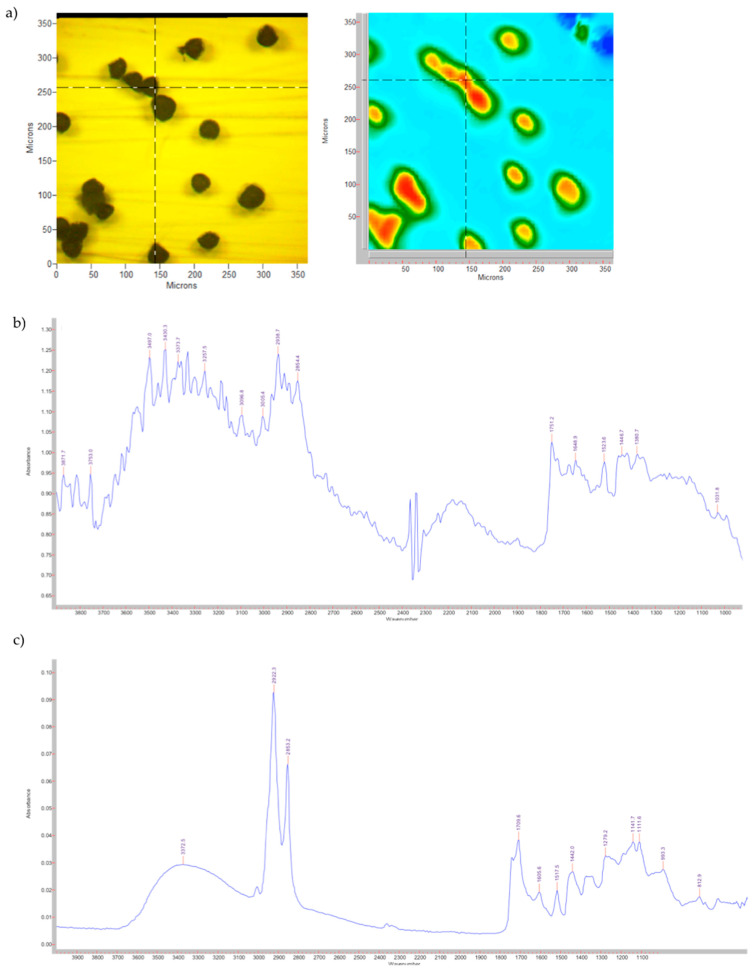
Untreated pollen grains. (**a**) Visible and FPA images; (**b**) spectrum obtained with the microscope; (**c**) spectrum obtained with the spectrometer.

In Figure 1, the visible image and the FPA image, together with the spectra obtained with the microscope and the spectrometer for the untreated pollen grains, are shown.

In accordance with the literature [32], the infrared spectrum allows the identification of the three main components of pollen based on the different vibrational bands:

1. Lipids: 1700–1760 cm^−1^ (C = O stretching), 1440–1450 cm^−1^ (CH_2_ deformation) and 1280 cm^−1^ (C – O stretching, not visible due to the lower resolution);
2. Proteins: 1600–1650 cm^−1^ (amide I) and 1515–1525 cm^−1^ (amide II);
3. Carbohydrates: 1000–1200 cm^−1^ (C – O – C and C – OH stretching).

In addition, there are two other bands attributable to the three components, ≈3300 cm^−1^ stretching of the – O – H and 2950–2850 cm^−1^ stretching of the aliphatic C – H.

The different resolutions between the two spectra reported in Figure 1b,c are correlated by the use of different ATR crystals (Ge vs. Zn/Se) and the major sensitivity of the ATR microscope to the humidity.

**Figure 2:**
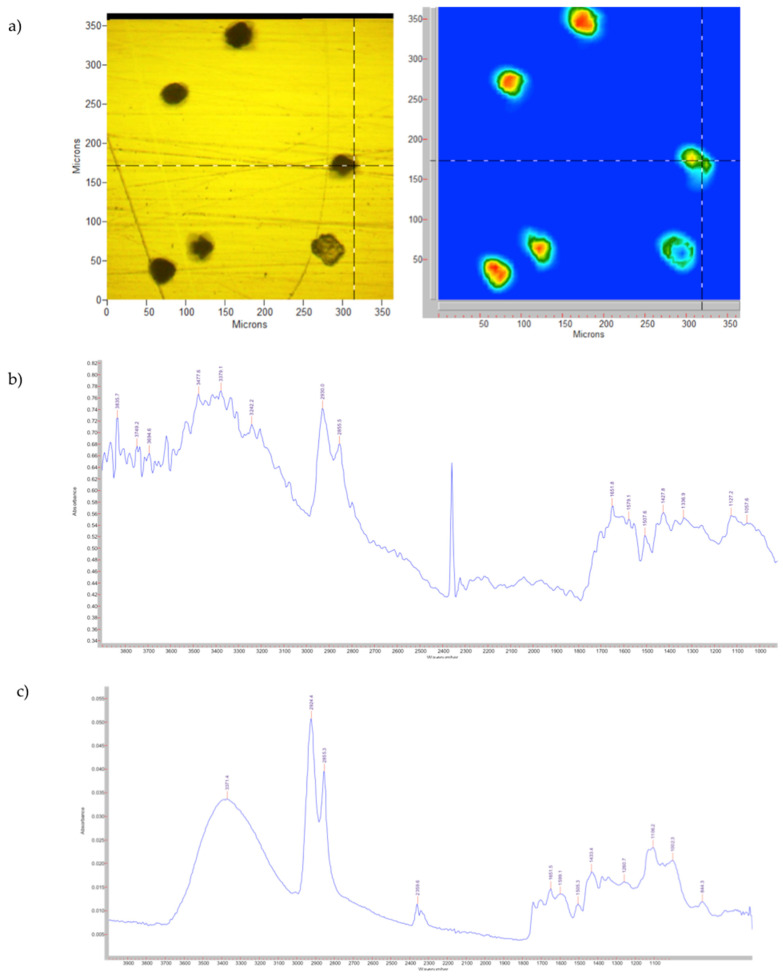
SPMs: (**a**) visible and FPA images; (**b**) spectrum obtained with the microscope; (**c**) spectrum obtained with the spectrometer.

If the data of the untreated pollen grains are compared with the ones obtained for SPMs, shown in Figure 2, the major highlighted differences are represented by a reduction in intensity of the bands corresponding to lipids and proteins. The carbohydrate bands appear to show a less marked reduction in their intensity. Unlike the literature [33], it is difficult to define a greater isolation of sporopollenin as its bands are almost superimposable to those of proteins.

**Figure 3:**
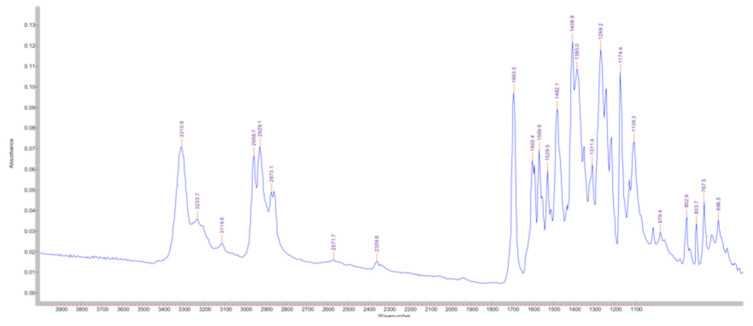
ETZ spectrum obtained with the spectrometer.

On the other hand, the infrared spectrum of the solar filter (Figure 3) shows four characteristic bands that differentiate it from pollen; ≈3300 cm^−1^ (N-H stretching of secondary amines), ≈1700 cm^−1^ (C = O stretching of esters), ≈1410 cm^−1^ (bending of C – H bonds) and ≈1270 cm^−1^ (C – N stretching of aromatic amines).

**Figure 4:**
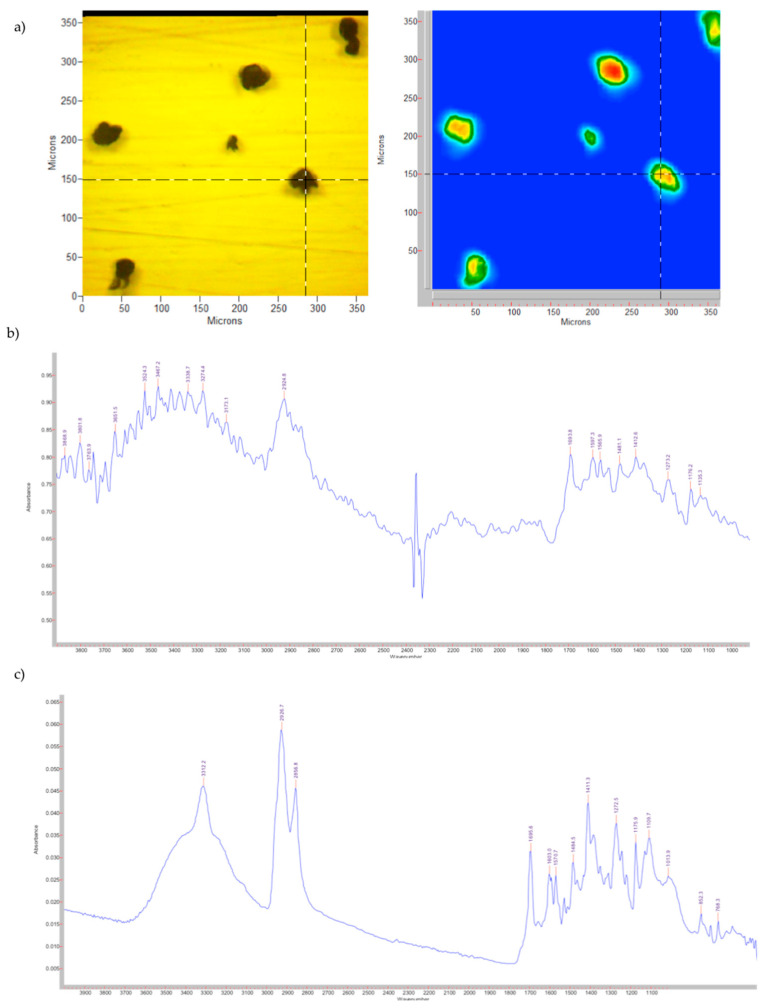
ETZ-SPMs before washing cycles: (**a**) visible and FPA images; (**b**) spectrum obtained with the microscope; (**c**) spectrum obtained with the spectrometer.

In Figure 4, the visible image and the FPA image of the ETZ-SPMs before the washing cycles necessary to remove the non-encapsulated solar filter, along with the spectra obtained with the microscope and the spectrometer, are shown.

The three characteristic bands of the solar filter are very evident in the spectrum obtained with the spectrometer, in particular the bands at ≈3300 cm^−1^ and ≈1700 cm^−1^, while with the microscope the band at ≈3300 cm^−1^ is more difficult to identify due to the sensitivity of the microscope to humidity, but the intense signal around 1700 cm^−1^ confirms the presence of the solar filter, suggesting that a non-negligible amount of ETZ is deposited outside the pollen grains.

**Figure 5:**
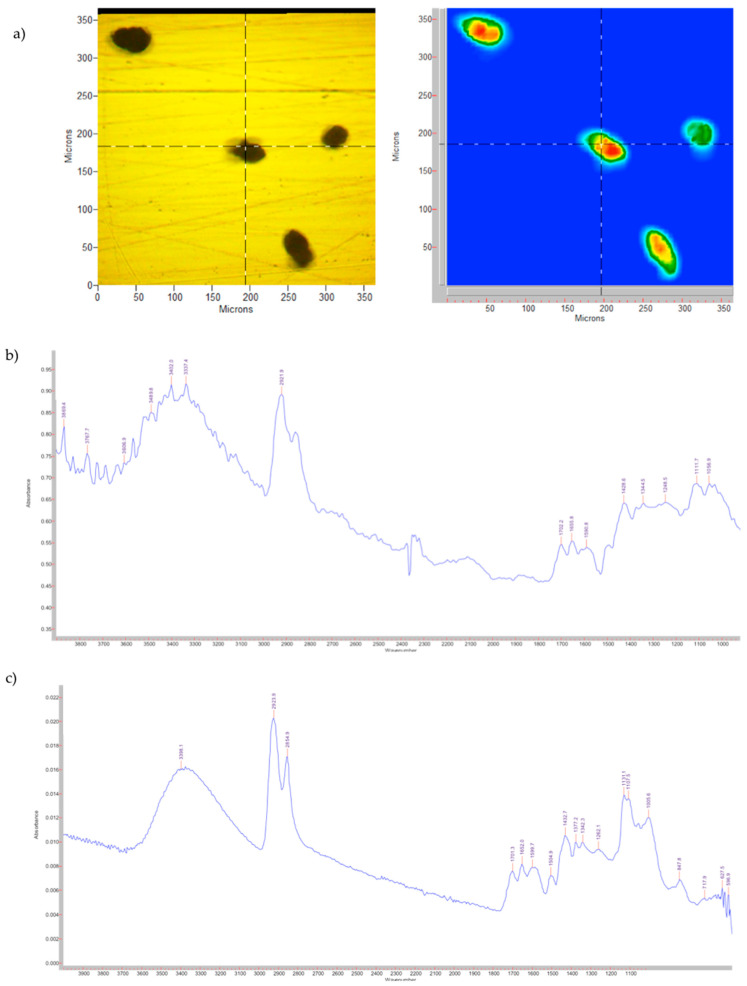
ETZ-SPMs after washing cycles: (**a**) visible and FPA images; (**b**) spectrum obtained with the microscope; (**c**) spectrum obtained with the spectrometer.

Conversely, when ETZ-SPMs were subjected to the procedure for the removal of the non-encapsulated filter, the band at ≈3300 cm^−1^ is no longer observable and the ester band at ≈1700 cm^−1^ together with other characteristic bands of the solar filter, such as the one at 1410 cm^−1^, has been significantly reduced, indicating that a substantial amount of sunscreen has been removed from outside the pollen (Figure 5).

Furthermore, the samples were also observed by using the optical microscope.

**Figure 6:**
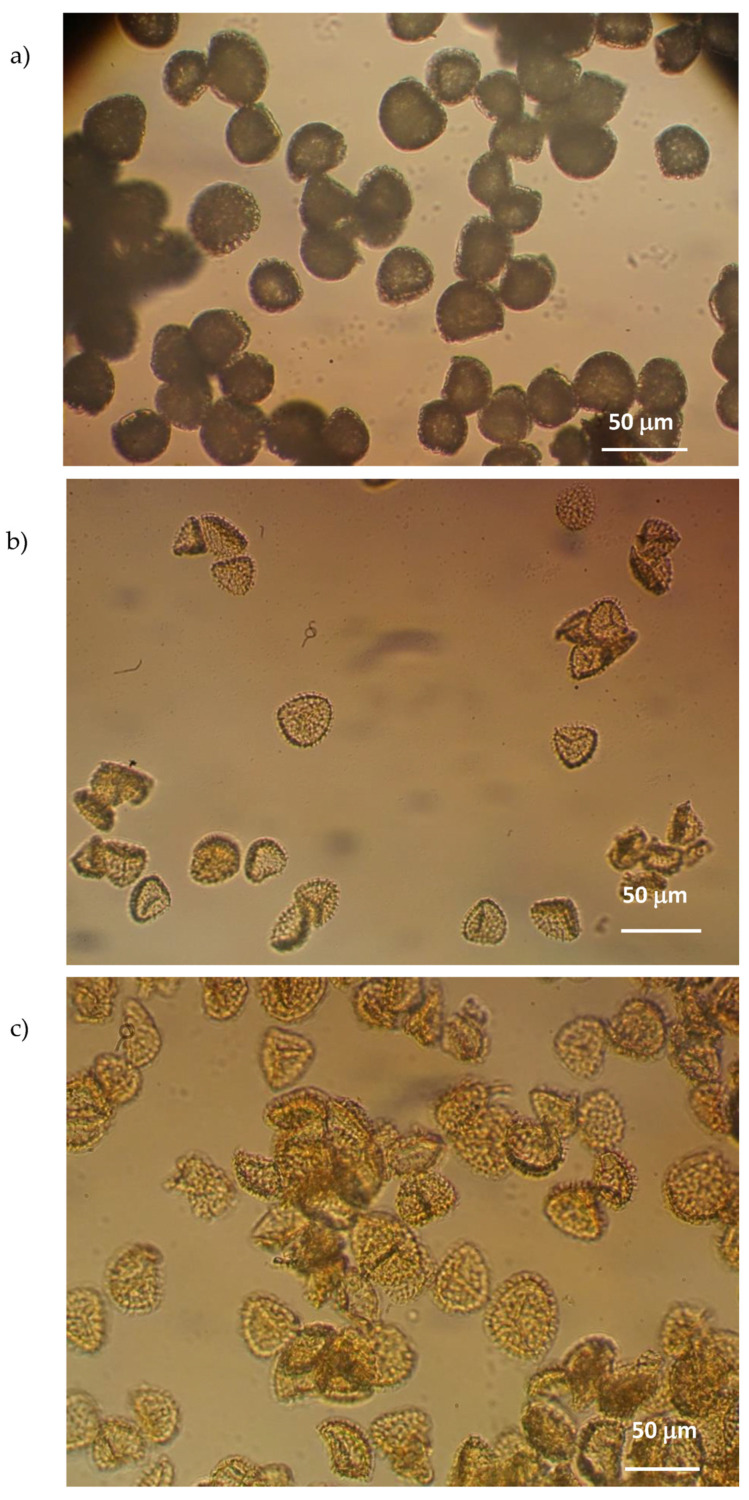
Photomicrographs of (**a**) untreated pollen grains, (**b**) SPMs and (**c**) ETZ-SPMs obtained by optical microscopy.

In Figure 6, the photomicrographs obtained for the natural pollen, the SPMs and the ETZ-SPMs are shown. In all cases, the size of the granules, of about 30 μm, confirms the literature data [34]. It is also interesting to note that both the emptying treatment and the filling procedure with the solar filter do not cause breakage of the granules or affect the homogeneity of the sample and its size.

### 3.3. Differential Scanning Calorimetry and Thermogravimetric Analysis

A further characterization of ETZ-SPMs was conducted by analyzing their thermal behavior using a differential scanning calorimeter (DSC), in order to verify the effective encapsulation of the solar filter within the microcapsules. Therefore, both SPMs and ETZ-SPMs were analyzed. For comparison, the ETZ alone and the physical mixture given by ETZ and empty SPMs in the same weight ratio present in the microcapsules loaded with solar filter were also analyzed.

**Figure 7:**
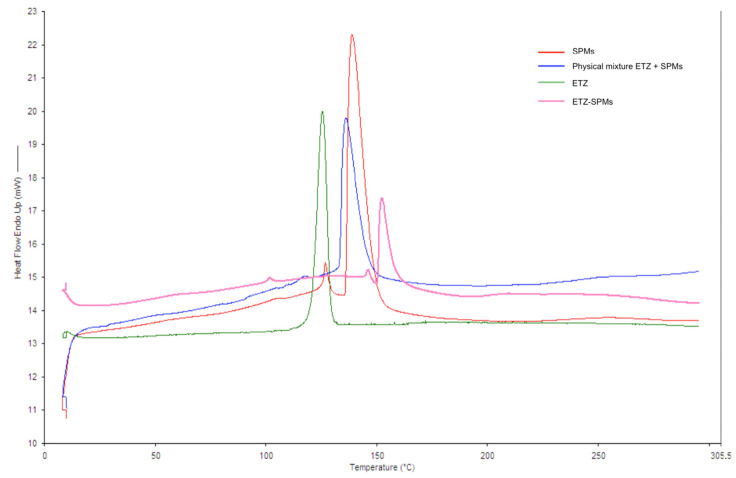
DSC of empty SPMs, ETZ, ETZ-SPMs and physical mixture ETZ + SPMs.

The thermograms obtained by DSC are shown in Figure 7. The melting peak at about 120 °C relative to ETZ confirms the literature data (123.27 °C) [35] and reflects its crystalline structure. It is possible to notice a shift at higher temperatures of the peak relative to the ETZ-SPMs compared to the empty SPMs. Furthermore, the melting peak of the solar filter disappears in the thermogram of ETZ-SPMs, indicative of a change from a crystalline state to a dispersed amorphous state. The physical mixture shows the peak at about 140 °C characteristic of sporopollenin microcapsules, but the spike disappears at about 120 °C and the ETZ melting peak is not detected, a sign of a surface interaction between the two products.

**Figure 8:**
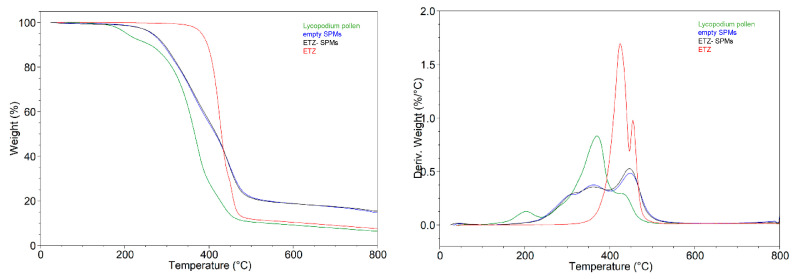
TG (**left**) and DTG (**right**) curves of *Lycopodium* pollen (green), empty SPMs (blue), ETZ-SPMs (black) and ETZ (red).

Thermograms of *Lycopodium* pollen grains, empty SPMs, ETZ and ETZ- SPMs (batch no. 6, Table 1) from thermogravimetric analysis are shown in Figure 8. As expected, empty SPMs show a higher thermal stability than the *Lycopodium* pollen grains with a starting degradation temperature (T_start_) of 265 °C and 172 °C, respectively. This is mainly due to the removal of the biological material within the pollen, which preserves the thermally stable exine and intine components. The ETZ compound shows higher thermal stability than empty SPMs, with a T_start_ value of 400 °C. On the other hand, the ETZ compound itself shows higher thermal stability than empty SPMs, with a T_start_ value of 400 °C and, for this reason, there is no significant effect of ETZ on the stability of the filled versus unfilled sporopollenin. In the temperature range around 400 °C, the difference in mass loss between the ETZ-loaded and empty sporopollenin is consistent with the previously determined EE% for the same batch (6.49 ± 0.28).

### 3.4. In Vitro Release Studies 

In vitro release studies through the dialysis membrane were carried out. In any case, the spectrophotometric analysis of the receiving phase samples did not show a detectable amount of ETZ for each performed withdrawal (LOQ = 0.0433 μg/mL). Previous studies reporting on the topical application of sporopollenin microcapsules suggested that the release of actives could only take place after applying a moderate pressure, such as that applied by spreading a formulation on the skin [19]. Therefore, it can be apparent that the ETZ-SPMs are stable in water solution at least for the 24 h of the in vitro release study, without allowing the solar filter to exit from the microcapsules. For further investigation, ETZ-SPMs were subjected to a stability study after having dispersed them in purified water or in lactate buffer at pH 5.5 for a week, in order to verify that the release of solar filter from the microcapsule in an aqueous environment did not occur, an important factor for the formulation development of the product in anticipation of a skin application. In no case was the characteristic peak of ETZ at the wavelength of maximum absorption of the solar filter (303 nm) revealed and therefore the product can be considered stable for the observed period.

### 3.5. SPF Determination

In order to evaluate a possible cosmetic application of ETZ-SPMs, the sun protection factor (SPF), which gives an indication of the filtering capacity of a solar product and provides a correct indication of the level protection and therefore of the duration of exposure to UVB radiation without having erythematogenic effects, was determined. The results obtained did not show a high protective capacity against UVB radiation by the SPMs, although ETZ-SPMs possessed a higher SPF with respect to empty SPMs with a statistically significant difference. Indeed, the mean SPF values measured were 1.20 ± 0.06 and 1.50 ± 0.09 for the empty SPMs and ETZ-SPMs, respectively; the values found are lower than the value of 3.4 calculated with the program Sunscreen-simulator (BASF) for the ETZ solar filter at the same concentration (1% *w*/*w*). This result seems to confirm that the solar filter is encapsulated inside the microcapsules and is not released in the aqueous medium of the formulation until use. 

The Cosmetic Regulation (EC) No. 1223/2009 also establishes that sunscreen products must also contain filters that are able to protect the skin from UVA radiation and therefore this feature has also been evaluated. In this case, the results reported in Table 3 and Table 4 show a UVA ratio in both cases ≥ 0.90 with the highest level of Star Rating (5 stars) definable as “ultra” protection, the highest level of protection according to the “Boots Star Rating System”. It can therefore be considered that the SPMs have a high protective power against UVA radiation.

### 3.6. Cutaneous Permeation and Distribution Studies

A further step was to verify the ability of the solar filter encapsulated in the SPMs to interact with the skin and permeate through excised porcine ear skin. The in vitro permeation experiments were performed using as donor phase two different amounts of ETZ-SPMs, namely 50 mg and 200 mg, containing about 2.7 and 10.6 mg of ETZ, respectively, to investigate the influence of the applied amount on both the permeation and the accumulation of the sun filter in the skin. In any case, the transcutaneous permeation of ETZ was observed throughout the 24 h experiment, during which the permeation was monitored. These data were quite expected, since it is widely known that ETZ is not prone to skin permeation due to its high molecular weight (823 Da) and log *p* > 8.10 [36,37]. Anyway, it is crucial to assess the role of the formulation in not enhancing the skin flux of a permeant. 

**Figure 9:**
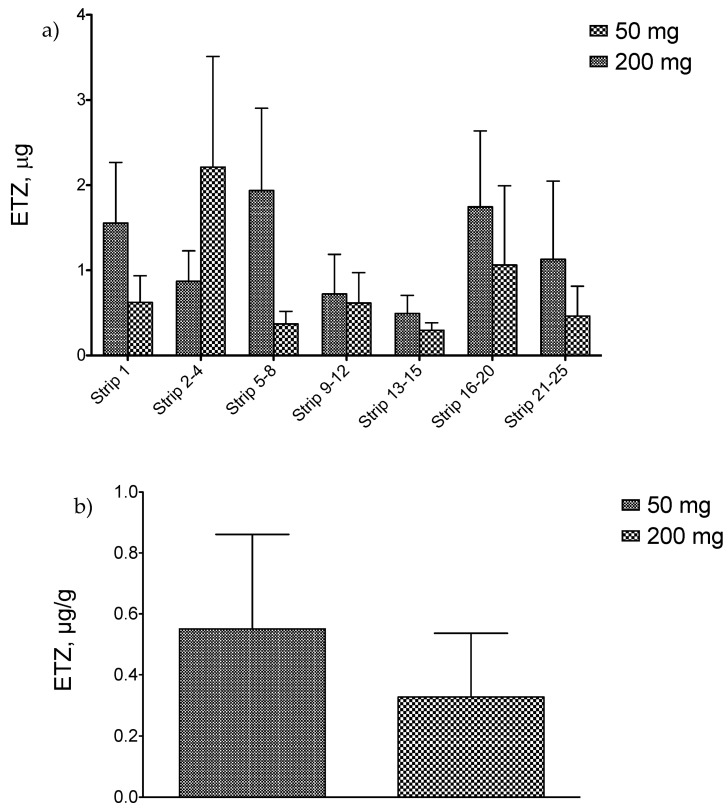
Recovery of ETZ (**a**) in the different tape strips (stratum corneum) and (**b**) in the skin depth (epidermis and dermis) when different amounts of ETZ-SPMs are applied on the porcine ear skin.

The in vitro penetration data after skin application of ETZ-SPMs in different amounts are reported in Table 5 and illustrated in Figure 9a,b for the recovery of ETZ in the tape strips (stratum corneum) and in the skin depth (epidermis and dermis), respectively. The obtained results showed that the application of either 50 or 200 mg of ETZ-SPMs produced a similar recovery of solar filter in all the skin layers, with a total amount of sun filter retained accounting for 6.195 µg and 8.782 µg, respectively.

It is evident that the amount of ETZ accumulated in the skin does not depend on the amount of filter applied, as there are no statistically significant differences between the results of the two tests. The observed behavior could be related to the finding reported above (Section 3.4) that the SPMs do not release the active ingredient in the aqueous medium, therefore only the preparation in direct contact with the skin allows the release of the filter for interaction of the sporopollenin with the skin itself, with an unknown mechanism that should be further elucidated. Degradation mechanisms of pollen grains are not yet fully understood, but it has been reported that *L. clavatum* microcapsules undergo degradation in human blood plasma and the release of the contents occurs through an enzymatic pathway [38,39,40]. Even though the literature lacks a characterization of the enzymatic species capable of degrading exine microcapsules [6], some speculation could be performed. Since it has been reported that the skin itself possesses a high enzymatic biotransformation activity [41,42], it can be hypothesized that some of the enzymes that are present in the skin (esterase, dehydrogenase, monooxygenase, etc.), and represent a further barrier together with the stratum corneum, could degrade the sporopollenin and allow ETZ release. 

Anyway, if we try to analyze the obtained results in terms of sun protection, we have to take into account that when we performed the SPF and UVA protection experiments, we applied 2 mg/cm^2^ of a 1% ETZ semisolid formulation, corresponding to ≅0.38 mg/cm^2^ ETZ-SPMs. In this context, it seems that the application of a dose of 50 mg ETZ-SPMs could be enough to exploit the property of the pollen grains in protecting from UVA radiation and to obtain a complete photoprotection. 

Moreover, the results obtained are consistent with the very few studies reported in the literature for ETZ investigating the skin penetration of the sun filter from different formulations at different concentrations of the active ingredient [36,43]. In particular, Ref. [43] studied the penetration profile of ETZ into rat skin or reconstituted skin from O/W and W/O emulsions containing 6% sun filter. In every case, the amount of ETZ penetrating into the first skin layers was of the same order of magnitude (a few micrograms) as in the present study, confirming our hypothesis that SPMs could represent an alternative to conventional formulations for sunscreen development.

## 4. Conclusions

In recent years, one of the most important challenges of cosmetic research has been to find innovative formulations that are functional and respectful of the environment at the same time. In this perspective, SPMs deriving from natural pollen, capable of encapsulating active ingredients by passive loading and therefore a method that is ecological, cost-effective, simple and relatively quick, can represent a valid alternative to conventional delivery systems. Furthermore, the possibility of functioning both as a release system and as an active ingredient provides these products with added value, together with easy availability and good reproducibility in the production phase.

In the present work, *L. clavatum* SPMs have been proposed as an alternative delivery system for a solar filter, ETZ, considered safe for both humans and the ecosystem; the data obtained from the imaging and FTIR analysis demonstrated the intact and monodisperse nature of the *L. clavatum* SPMs and the removal of foreign materials from the microcapsule. Moreover, the investigations on the thermal behavior of ETZ-SPMs confirmed that the solar filter was successfully loaded into the microcapsules and the aqueous dispersion remain stable over time, supporting the idea that SPMs may represent a promising material for the encapsulation of active ingredients that need to be protected from the external environment and subsequently released in the tissues of interest. Additionally, ETZ-SPMs are able to deliver the solar filter to the first layers of the skin in order to let ETZ protect from UVB light, without permeating through the skin as requested by the Cosmetic Regulation. Finally, for the first time in the literature, *L. clavatum* SPMs have been demonstrated to possess a very high UVA protection at the concentration of use in the cosmetic field, accordingly with the *Boots Star Rating* and the *Star Category* based on the *UVA ratio* parameter.

Therefore, the results of this work seem very interesting for the cosmetic field and can be prodromal for future studies on the use of sporopollenin microcapsules from different plant species, with the dual function of vehicle and active ingredient with photoprotective properties.

## Figures and Tables

**Table 1:** *Star Category* and *Star Rating values* according to the UVA ratio (2004 *revision*).

UVA Ratio	Star Category	Star Rating
0.0 to 0.19	-	Too low for UVA claim
0.2 to 0.39	*	Minimum
0.4 to 0.59	**	Moderate
0.6 to 0.79	***	Good
0.8 to 0.89	****	Superior
≥0.90	*****	Ultra

**Table 2:** Physical–chemical characterization of ETZ-SPMs produced with acetone and four consecutive centrifugations with solvent replacement after supernatant removal (*n* = 2).

Batch	ETZ, mg/g_SPMs_	EE%
1	61.28 ± 0.39	11.91 ± 0.33
2	59.44 ± 0.37	10.39 ± 0.38
3	43.34 ± 4.32	7.76 ± 0.77
4	62.12 ± 8.56	11.34 ± 1.58
5	55.82 ± 7.46	9.93 ± 1.33
6	36.99 ± 1.60	6.49 ± 0.28

**Table 3:** Calculation of SPF and UVA ratio of empty SPMs (transpore 2 mg/cm^2^).

Mean	STD	COV (%)	UVA/UVB Ratio	Star Category	Λ_cr_ (nm)
1.23	0.01	0.42	1.33	Ultra	390
1.22	0.01	0.48	1.33	Ultra	390
1.23	0.01	0.55	1.37	Ultra	390
1.10	0.01	1.31	1.94	Ultra	392
1.22	0.01	1.02	1.39	Ultra	391
**SPF (mean ± SD): 1.20 ± 0.06**					

**Table 4:** Calculation of SPF and UVA ratio of ETZ-SPMs (transpore 2 mg/cm^2^).

Mean	STD	COV (%)	UVA/UVB Ratio	Star Category	Λ_cr_ (nm)
1.46	0.02	1.40	1.06	Ultra	389
1.66	0.02	1.46	1.02	Ultra	389
1.46	0.01	0.80	1.07	Ultra	389
1.49	0.00	0.21	1.07	Ultra	389
1.46	0.01	0.30	1.11	Ultra	390
**SPF (mean ± SD): 1.50 ± 0.09**					

**Table 5:** Recovery of ETZ in both the stratum and the skin depth, when different amounts of ETZ-SPMs are applied on the porcine ear skin (mean ± standard error).

Skin Depth	ETZ (µg) 24 h	
	Donor Phase 50 mg	Donor Phase 200 mg
***Stratum corneum***		
Strip 1	0.624 ± 0.312	1.554 ± 0.713
Strip 2–4	2.212 ± 1.300	0.872 ± 0.357
Strip 5–8	0.370 ± 0.148	1.937 ± 0.966
Strip 9–12	0.617 ± 0.355	0.722 ± 0.465
Strip 13–15	0.295 ± 0.088	0.494 ± 0.212
Strip 16–20	1.063 ± 0.931	1.745 ± 0.893
Strip 21–25	0.463 ± 0.351	1.131 ± 0.918
***Viable epidermis/dermis***	0.551 ± 0.310	0.327 ± 0.210
